# Supplementary material for: The Tip60/Ep400 chromatin remodeling complex impacts basic cellular functions in cranial neural crest-derived tissue during early orofacial development
Source: Int J Oral Sci. 2023 Apr 6;15:16. doi: 10.1038/s41368-023-00222-7 (PMC10079831; doi:10.1038/s41368-023-00222-7)
Supplement: Supplementary file 1 — Supplemental Material [file 41368_2023_222_MOESM1_ESM.pdf]

Supplemental Material

The Tip60/Ep400 chromatin remodeling complex impacts basic cellular functions in cranial neural crest-derived tissue during early orofacial development

Sebastian Gehlen-Breitbach[1]*, Theresa Schmid[1,2]*, Franziska Fröb[1], Gabriele Rodrian[2], Matthias Weider[2], Michael Wegner[1#], Lina Gölz[2#]

[1]Institut für Biochemie, Emil-Fischer-Zentrum, Friedrich-Alexander-Universität Erlangen-Nürnberg, D-91054 Erlangen, Germany, [2]Zahnklinik 3 -Kieferorthopädie, Universitätsklinikum Erlangen und Friedrich-Alexander-Universität Erlangen-Nürnberg, D-91054 Erlangen, Germany

* These authors should both be considered as co-first authors.

[#] Corresponding authors

Correspondence to: Prof. Dr. Lina Gölz (lina.goelz@uk-erlangen.de)

or Prof. Dr. Michael Wegner (michael.wegner@fau.de)

Institut für Biochemie, FAU Erlangen-Nürnberg

Fahrstrasse 17, D-91054 Erlangen, Germany

phone: +49 9131 8524620; fax: +49 9131 8522484

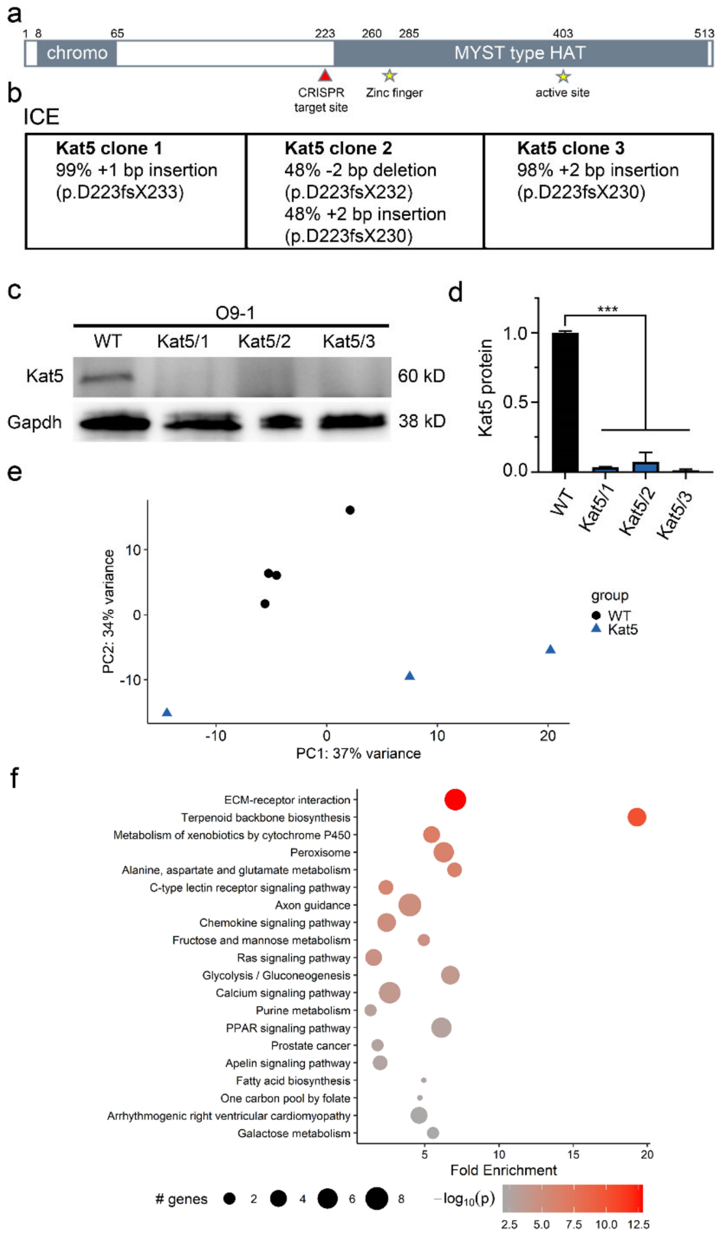

Supplemental Figure 1: CRISPR/Cas9-dependent Kat5 inactivation in O9-1 cells. (a) Schematic representation of the Kat5 protein, its domains (grey boxes) and structural features (asterisks) as well as the target site of the CRISPR/Cas9 editing (red arrowhead). Numbers above the bar correspond to positions of amino acid residues. Chromo, chromodomain; HAT, histone acetyltransferase domain. **(b)** Characterization of the CRISPR/Cas9-evoked gene editing events in Kat5 ko clones as determined by ICE. **(c,d)** Detection of Kat5 and Gapdh proteins in wildtype O9-1 cells (WT) and gene-edited clones by Western blotting (c) and quantification of Kat5 levels (d) relative to the amount of total protein (n=3). The size of detected proteins in kDa (left) was estimated from co-electrophoresed marker proteins. **(e)** PCA-plot of RNA sequencing samples from gene-edited clones (n=3) and wildtype cells (n=4). **(f)** KEGG clustering of genes down-regulated following Kat5 inactivation to identify gene ontology pathways and processes affected by Kat5 inactivation. Statistical significance was determined by one-way ANOVA and Dunnett's post test (***, P ≤0.001).

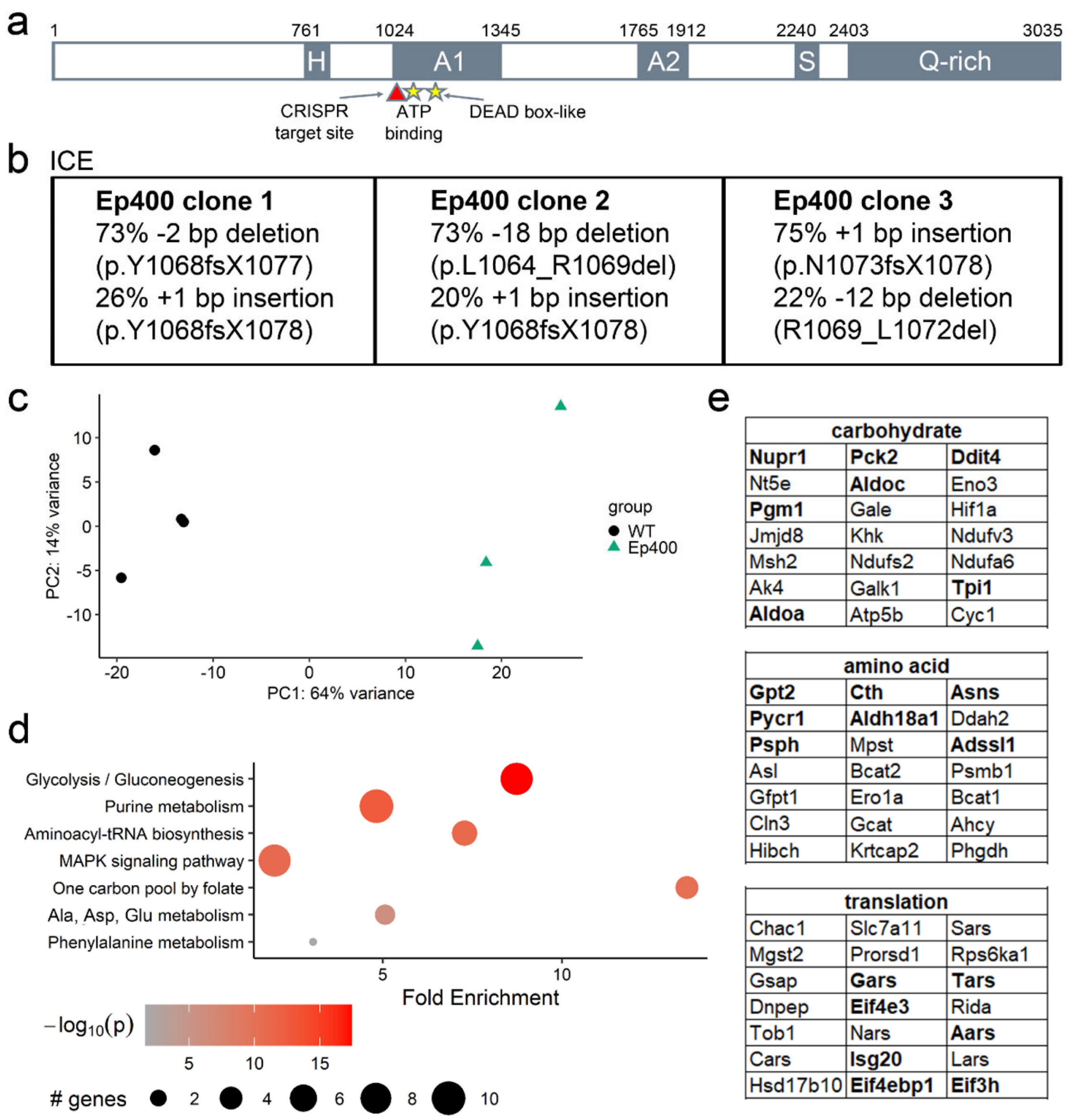

Supplemental Figure 2: CRISPR/Cas9-dependent Ep400 inactivation in O9-1 cells. (a) Schematic representation of the Ep400 protein, its domains (grey boxes) and structural features (asterisks) as well as the target site of the CRISPR/Cas9 editing (red arrowhead). Numbers above the bar correspond to positions of amino acid residues. H, HSA domain; A1 A2, parts of the bipartite ATPase domain; S, SANT domain; Q-rich, glutamine-rich domain. **(b)** Characterization of the CRISPR/Cas9-evoked gene editing events in Ep400 ko clones as determined by ICE. **(c)** PCA-plot of RNA sequencing samples from gene-edited clones (n=3) and wildtype cells (n=4). **(d)** KEGG clustering of genes down-regulated following Ep400 inactivation to identify gene ontology pathways and processes affected by Ep400 inactivation. **(e)** Selection of top downregulated genes shared between Ep400 and Kat5 ko clones and sorted by category (carbohydrate metabolism, amino acid metabolism, protein biosynthesis). Genes studied in validation experiments are in bold.

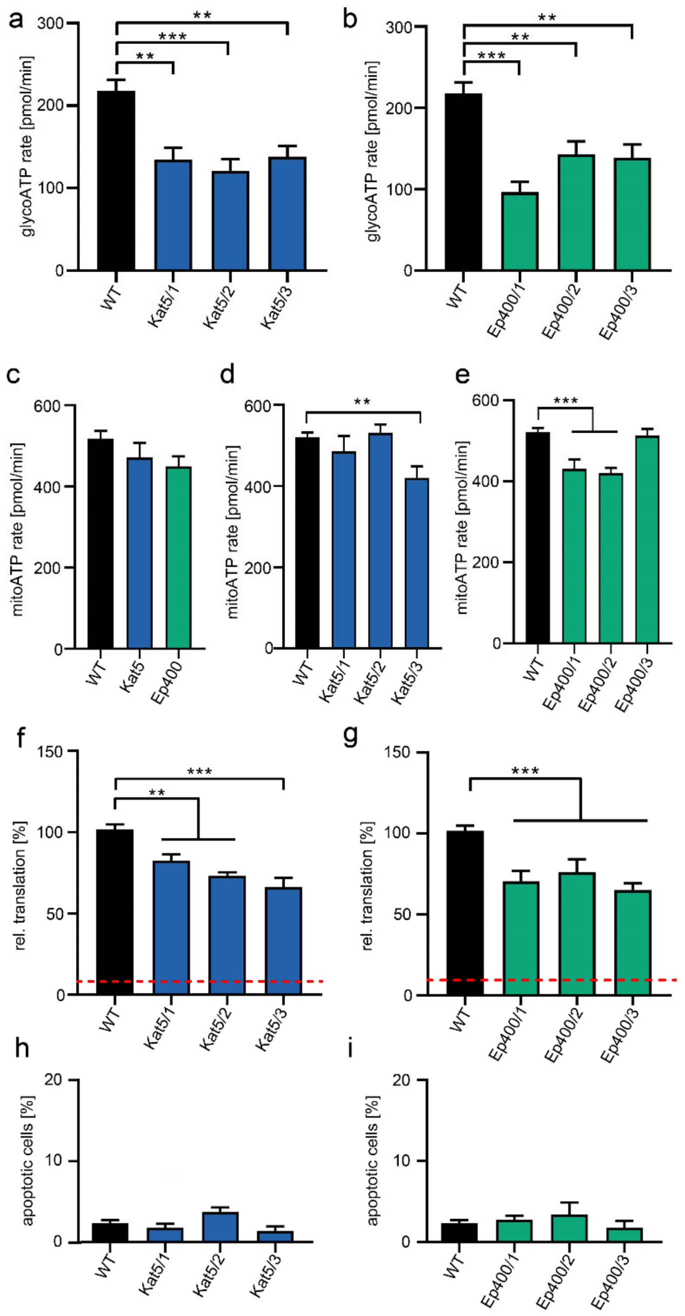

Supplemental Figure 3: Consequences of Kat5 and Ep400 inactivation on ATP generation, translation and apoptosis in cultured O9-1 cells. (a,b) Determination of glycolytic ATP generation rates (in pmol per minute) in single clones with Kat5 (a, blue bars) or Ep400 (b, green bars) inactivation relative to wildtype (WT, black bars) cells. **(c-e)** Determination of mitochondrial ATP generation rates (in pmol per minute) averaged over three clones with Kat5 or Ep400 inactivation (c), or determined in single clones (d, for Kat5 ko clones; e, for Ep400 ko clones) relative to wildtype cells. **(f,g)** OPP incorporation in nascent transcripts (with levels in WT cells set to 100%) in single clones with Kat5 (f) or Ep400 (g) inactivation. Red lines correspond to OPP incorporation in the presence of cycloheximide. **(h,i)** Percentage of cleaved caspase 3-positive cells in cultures of single clones with Kat5 (h) or Ep400 (i) inactivation relative to wildtype cells. Statistical significance was determined by one-way ANOVA and Dunnett’s post test (*, P ≤0.05; **, P ≤0.01; ***, P ≤0.001).

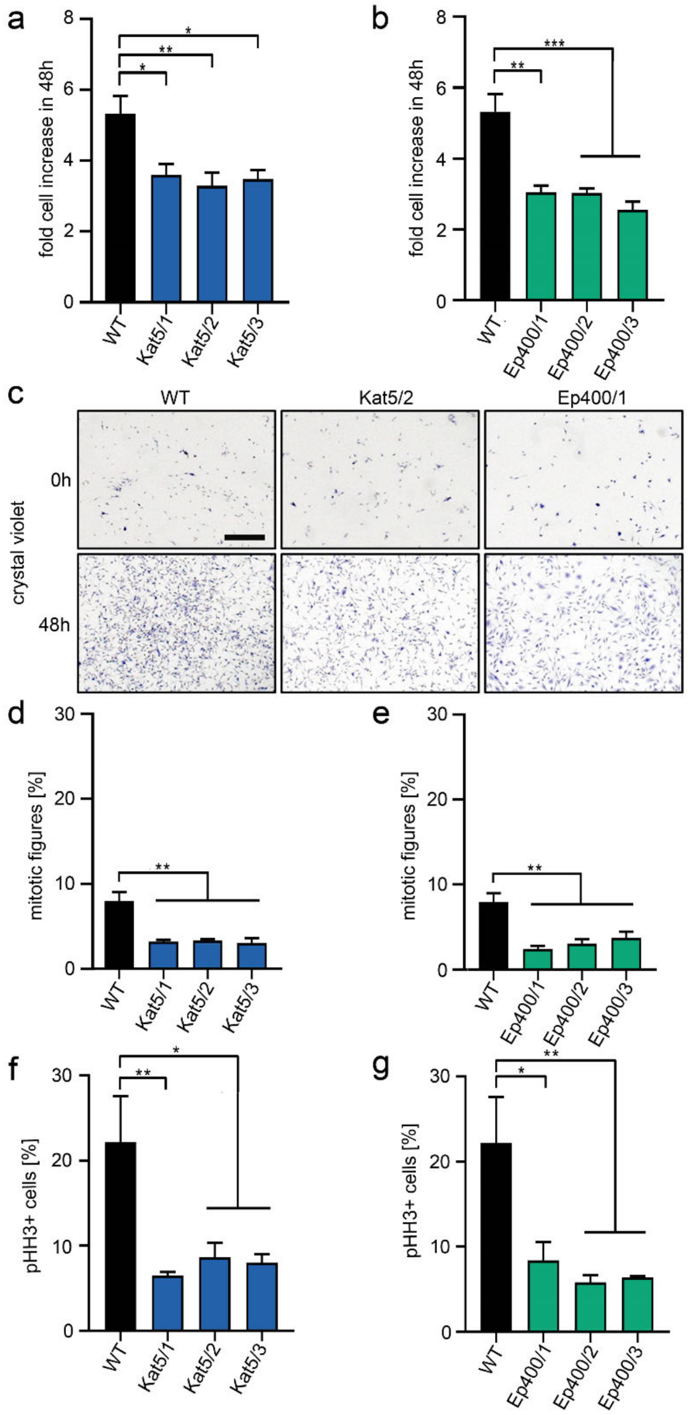

Supplemental Figure 4: Consequences of Kat5 and Ep400 inactivation on proliferation in cultured O9-1 cells. (a,b) Determination of 48 h proliferation rates in single clones with Kat5 (a, blue bars) or Ep400 (b, green bars) inactivation relative to wildtype (WT, black bars) cells as determined by crystal violet staining. Staining levels at 0 h were set to 1. **(c)** Representative images of crystal violet stainings of cultured wildtype, clone Kat5/2 and clone Ep400/1 cells at 0 h and 48 h. Scale bar: 400 µm. **(d-g)** Percentage of mitotic (d,e) and phosphohistone H3-positive (f,g) cells in cultures of single clones (d,f, for Kat ko clones; e,g, for Ep400 ko clones) relative to wildtype cells. Statistical significance was determined by one-way ANOVA and Dunnett's post test (*, P ≤0.05; **, P ≤0.01; ***, P ≤0.001).

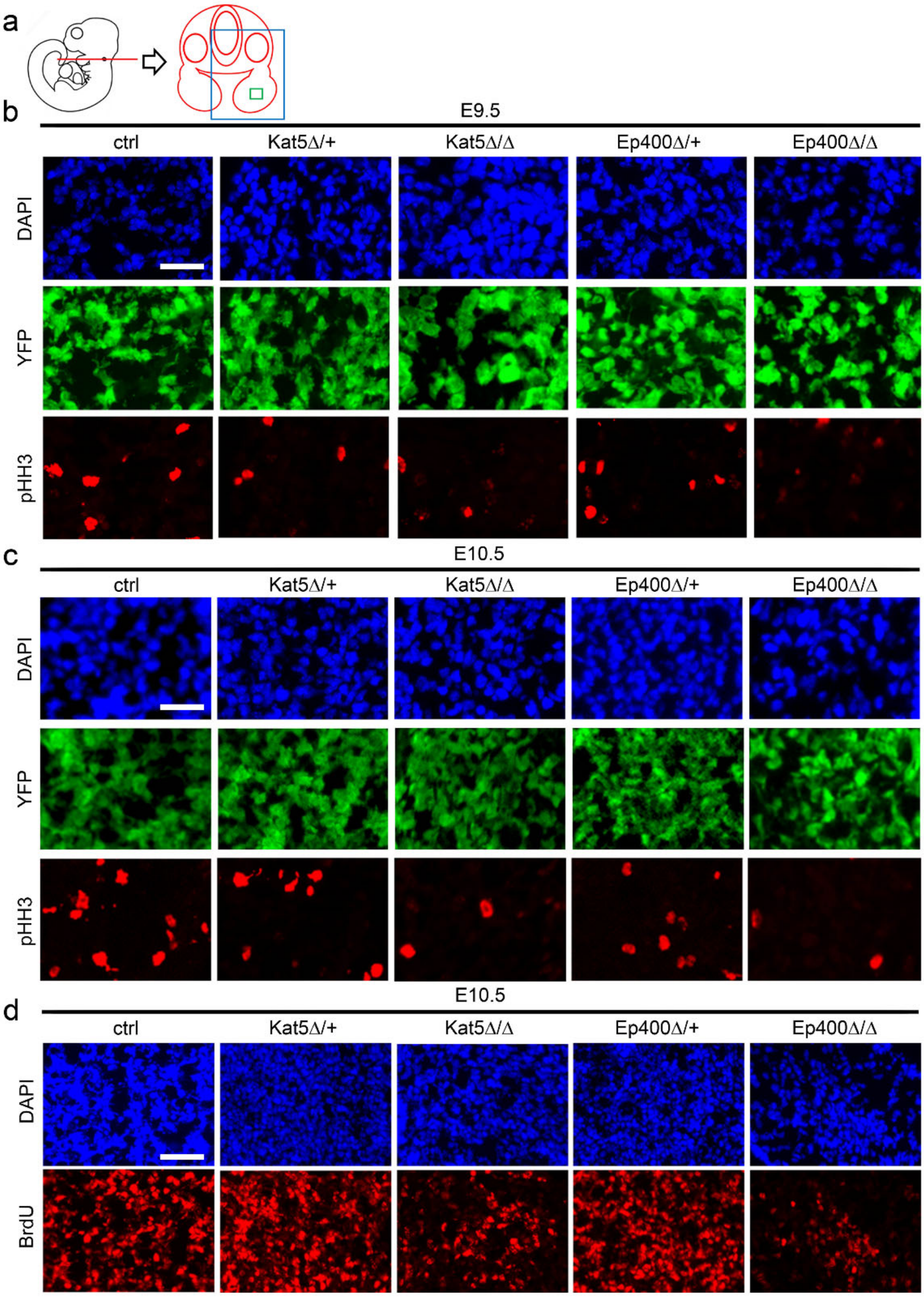

Supplemental Figure 5: Consequences of Kat5 and Ep400 inactivation on cranial neural crest proliferation. (a) Schematic representation of early mouse embryo and section used for analyses (red). The larger boxed area (blue) is depicted in Fig. 6c, the contained smaller area (green) in Suppl. Figs 5b-d and 6.a,b **(b-d)** Representative images of immunohistochemical stainings with anti-phosphohistone H3 (b,c, pHH3, red, bottom row) or anti-BrdU antibodies (d, red, bottom row) of transverse sections of the first pharyngeal arch from control (ctrl) embryos and age-matched heterozygous as well as homozygous embryos with Kat5 (Kat5Δ/+, Kat5Δ/Δ) or Ep400 (Ep400Δ/+, Ep400Δ/Δ) deletions at E9.5 (b) or E10.5 (c,d). DAPI counterstains are shown in the top row (blue), the neural crest-specific YFP autofluorescence in the middle row (green). Scale bar: 50 µm (b,c), 100 µm (d).

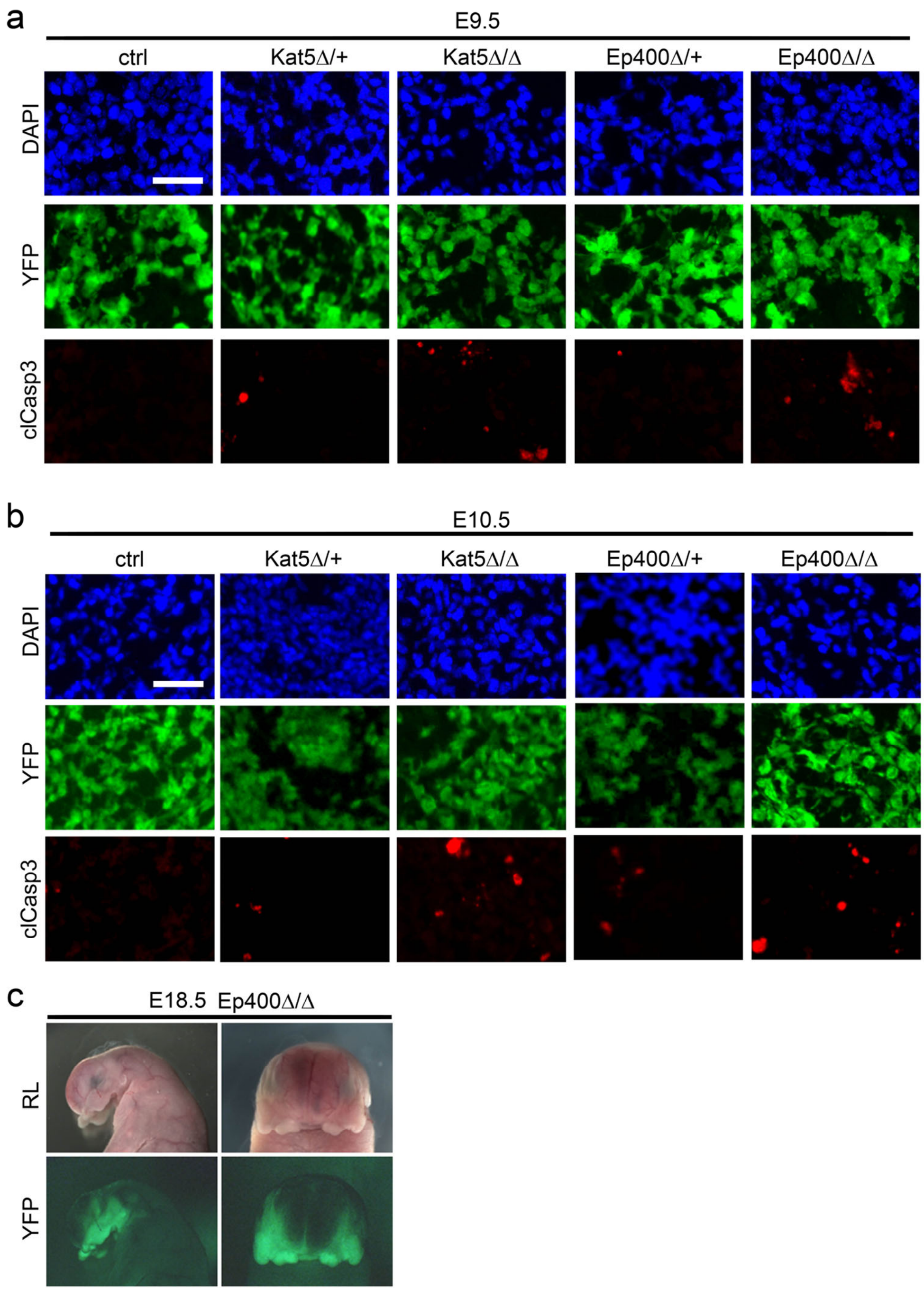

Supplemental Figure 6: Consequences of Kat5 and Ep400 inactivation on cranial neural crest survival. (a,b) Representative images of immunohistochemical stainings with anti-cleaved caspase 3 antibodies (red, bottom row) of transverse sections of the first pharyngeal arch from control (ctrl) embryos and age-matched heterozygous as well as homozygous embryos with Kat5 (Kat5Δ/+, Kat5Δ/Δ) or Ep400 (Ep400Δ/+, Ep400Δ/Δ) deletions at E9.5 (a) or E10.5 (b). DAPI counterstains are shown in the top row (blue), the neural crest-specific YFP autofluorescence in the middle row (green). Areas for presentation were as in Suppl. Fig. 5a. Scale bar: 50 µm. **(c)** Reflected-light microscopic (RL, upper row) and YFP-autofluorescent (lower row) images of heads from homozygous embryos with Ep400 (Ep400Δ/Δ) deletions at E18.5 (for comparison to ctrl and Kat5Δ/Δ embryos, see Fig. 7a.

Supplemental Table 1: List of oligonucleotides for genome-editing (CRISPR), ICE analysis, sequencing and quantitative PCR (qPCR) of chromatin precipitates (ChIP)

Name		Sequence	Application
Ep400 gRNA	sense<br>antisense	CACCGCTGGTTGGCCAAGCTATAC<br>AAACGTATAGCTTGGCCAACCAGC	Ep400 CRISPR guide sequence
Kat5 gRNA	sense<br>antisense	CACCGCCGGGTGACAATGTCGTCG<br>AAACCGACGACATTGTCACCCGGC	Kat5 CRISPR guide sequence
pX330 Seq		GGCCTATTTCCCATGATTCCTTC	sequencing of pX330 plasmids
Ep400 ICE	forward<br>reverse	GAAGACTCTGGGAAGAATGG<br>CTCCGTTCTCATTGACTCAC	ICE Ep400 amplicon generation
Kat5 ICE	forward<br>reverse	CTTGCAGGAAGAAGCCAATG<br>TGACAGTGACCTCTCAGGAAAG	ICE Kat5 amplicon generation
Pck2 TSS	forward<br>reverse	CTTCCCCGCCTTCCTTGTC<br>CCCGGGCTATGGGTCACT	ChIP qPCR of Pck2
Aldh18a1 TSS	forward<br>reverse	TGAGGGAGACGGTCTTTGTTG<br>TCGAGCACCCAGAAAGGAAC	ChIP qPCR of Aldh18a1
Asns TSS	forward<br>reverse	CTCCGCATCCGAACTCCC<br>TCCAACCGGTCTTGTCACTG	ChIP qPCR of Asns
Aars TSS	forward<br>reverse	TTGTGCGCATCTGAGGGAG<br>CGCCGGCTCTTCTCGTAG	ChIP qPCR of Aars
Eif4ebp1 TSS	forward<br>reverse	CGGGGACTACAGCACCAC<br>GCACGTGAGACTCCGGATC	ChIP qPCR of Eif4ebp1
Fasl TSS	forward<br>reverse	TGGCGTCTCTGTGCTAACTG<br>CAGAGCTGCGGAAGAGCTAA	ChIP qPCR of Fasl
Gm5741 TSS	forward<br>reverse	CTGTCCCAACCCTCACCTTT<br>ATGTCTAGCAAGGTGGCCAC	ChIP qPCR of Gm5741

Supplemental Table 2: List of oligonucleotides for RT-PCR

Name		Sequence	RT-PCR of …..
RT-PCR Ep400	forward<br>reverse	CTGGACTGGTTGGCCAAGCTA<br>GGTCCACTCCTGTCTCTTTG	Ep400
RT-PCR Kat5	forward<br>reverse	GTCTGACCGGAGCCACGACG<br>CAAGTGGCGTTGCAGACACTTG	Kat5
Aldoa	forward<br>reverse	AGGAGGAGTACATCAAGCGC<br>GCTCTGGTTAGTAGGCATGGT	Aldoa
Aldoc	forward<br>reverse	TCATAGGAGGAAACAGGCTTCA<br>CGATTCCAATTCGAGCGATTGA	Aldoc
Ddit4	forward<br>reverse	TGGGGTGGGTGTTAGGATCA<br>AGCCCCTCTCCTCACAGTT	Ddit4
Nupr1	forward<br>reverse	AAGTGAGGTTCTGGAGAGGG<br>AGTTCTTTTGGGGGCTGTCT	Nupr1
Pck2	forward<br>reverse	TCCAAACTCACTGTGCAGCT<br>TGAAATGCAAATGGTACTCTGACA	Pck2
Pgm1	forward<br>reverse	CTGCACCTGTGTCTGTTCCT<br>GGAGATGGCCTTGTTGCTGA	Pgm1
Tpi1	forward<br>reverse	ATAAACACCTGGGCCTGAGC<br>TGAGATGTATTGTCACAACCTGT	Tpi1
Adssl1	forward<br>reverse	GCTTGAAATTGTTGAGCCCTACA<br>ACATGGCTCCAACTGAACACA	Adssl1
Aldh18a1	forward<br>reverse	GTTGGAATCTGAGCATCACGC<br>CTTTGTCCTGGAGGTCGAGAA	Aldh18a1
Asns	forward<br>reverse	CCTGGTTCAAGATTTTGCAGGA<br>CTGGGTAATGGCGTTCAAAGA	Asns
Cth	forward<br>reverse	CCCTTAAGAACTGTCTTCTGTTTATCT<br>AGACAACATGATCCCAAAACAGA	Cth
Gpt2	forward<br>reverse	ACTGGGATTGTGCCACCATT<br>AACCCAACCCATCGCTTCTC	Gpt2
Psph	forward<br>reverse	GCAATGTGATCAGGCAGCAG<br>CCAGGAGCCTAACTGTGTGG	Psph
Pycr1	forward<br>reverse	ATAGCTTGCCCAGGGAGATG<br>TGGCTCTTGAATCTGCCCAT	Pycr1
Aars	forward<br>reverse	TGAGCCCATTCATGTCAGCA<br>TCCTCTGCAGACCTAAAGCC	Aars
Eif3h	forward<br>reverse	ACTGCCAGAACATCAAGGAGT<br>ACAGTCTGTCTACTCAACGCC	Eif3h
Eif4e3	forward<br>reverse	TCTGACAGATTAGCACAAAGGA<br>ACATACACGCAAAATTTGAACATGT	Eif4e3
Eif4ebp1	forward<br>reverse	TAGGCTGGGTTACCTGGGAA<br>TCATGTCGTCATTTCGAGTGGA	Eif4ebp1
Gars	forward<br>reverse	AGAGACTGGCAAGAAGGAGAC<br>GGGAGTAAAACCAAGCAGCC	Gars
Isg20	forward<br>reverse	CTCTGTGGAAGATGCCAGGG<br>AGAACTTGAGGAGTGGCAGC	Isg20
Tars	forward<br>reverse	AGACCTTGTACACCGGGAAG<br>GGTTCTCTTAACCCCCATCTACA	Tars

Supplemental Table 3: Antibodies for immunohistochemistry (IHC), immunocyto-chemistry (ICC), chromatin immunoprecipitation (ChIP) and Western blotting (WB)

Antibody	Application	Dilution	Source
rabbit anti-Ep400	ICC<br>IHC	1:2500<br>1:1000	Elsesser et al. 2019 (PMID: 32266943)
rabbit anti-Kat5	WB<br>ICC	1:1000<br>1:1000	Proteintech
rabbit anti-pHH3	ICC<br>IHC	1:2500<br>1:1000	Upstate Biotechnology
rabbit anti-cleaved Caspase3	IHC	1:200	Cell Signaling
rabbit anti-Sp7	IHC	1:500	Abcam
rabbit anti-H4K5,8,12,16ac	ChIP	2 µg/ChIP	Diagenode
rabbit anti-H2A.Z	ChIP	2 µg/ChIP	Diagenode
rabbit IgG	ChIP	2 µg/ChIP	Sigma-Aldrich
guinea pig anti-Sox6	IHC	1:1000	Stolt et al. 2006 (PMID: 17084361)
chicken anti-GFP	IHC	1:5000	Aves Labs
rat anti-BrdU	IHC	1:20	Abcam
donkey anti-rabbit-Cy3	ICC<br>IHC	1:500<br>1:200	Jackson ImmunoResearch
donkey anti-rat-Cy3	IHC	1:200	Jackson ImmunoResearch
donkey anti-chicken-Alexa488	IHC	1:1000	Jackson ImmunoResearch
